# Distinct and rich assemblages of giant viruses in Arctic and Antarctic lakes

**DOI:** 10.1093/ismeco/ycae048

**Published:** 2024-03-29

**Authors:** Thomas M Pitot, Josephine Z Rapp, Frederik Schulz, Catherine Girard, Simon Roux, Alexander I Culley

**Affiliations:** Department of Biochemistry, Microbiology and Bioinformatics, Université Laval, 2325 rue de l’Université, Québec, QC G1V0A6, Canada; DOE Joint Genome Institute, Lawrence Berkeley National Laboratory, 1 Cyclotron Road, Berkeley, CA 94720, United States; Center for Northern Studies, Université Laval, 2325 rue de l’Université, Québec, QC G1V0A6, Canada; IBIS Institute of Integrative Biology and Systems, Université Laval, 2325 rue de l’Université, Québec, QC G1V0A6, Canada; Center for Northern Studies, Université Laval, 2325 rue de l’Université, Québec, QC G1V0A6, Canada; Department of Biology, Université Laval, 2325 rue de l’Université, Québec, QC G1V0A6, Canada; DOE Joint Genome Institute, Lawrence Berkeley National Laboratory, 1 Cyclotron Road, Berkeley, CA 94720, United States; Center for Northern Studies, Université Laval, 2325 rue de l’Université, Québec, QC G1V0A6, Canada; Département des Sciences Fondamentales, Université du Québec à Chicoutimi (UQAC), Chicoutimi, QC G7H 2B1, Canada; DOE Joint Genome Institute, Lawrence Berkeley National Laboratory, 1 Cyclotron Road, Berkeley, CA 94720, United States; Department of Biochemistry, Microbiology and Bioinformatics, Université Laval, 2325 rue de l’Université, Québec, QC G1V0A6, Canada; Center for Northern Studies, Université Laval, 2325 rue de l’Université, Québec, QC G1V0A6, Canada; Pacific Biosciences Research Center, 1800 East-West Road, Honolulu, HI 96822, United States

**Keywords:** lacustrine polar virology, Nucleocytoviricota, meta-analysis, Last Ice Area margin, metagenomics

## Abstract

Giant viruses (GVs) are key players in ecosystem functioning, biogeochemistry, and eukaryotic genome evolution. GV diversity and abundance in aquatic systems can exceed that of prokaryotes, but their diversity and ecology in lakes, especially polar ones, remain poorly understood. We conducted a comprehensive survey and meta-analysis of GV diversity across 20 lakes, spanning polar to temperate regions, combining our extensive lake metagenome database from the Canadian Arctic and subarctic with publicly available datasets. Leveraging a novel GV genome identification tool, we identified 3304 GV metagenome-assembled genomes, revealing lakes as untapped GV reservoirs. Phylogenomic analysis highlighted their dispersion across all *Nucleocytoviricota* orders. Strong GV population endemism emerged between lakes from similar regions and biomes (Antarctic and Arctic), but a polar/temperate barrier in lacustrine GV populations and differences in their gene content could be observed. Our study establishes a robust genomic reference for future investigations into lacustrine GV ecology in fast changing polar environments.

## Introduction

Formally characterized in 2003 by Bernard La Scola *et al*., giant viruses (GVs) or *Nucleocytoviricota* challenged the traditional definition of a virus and blurred the line between viruses and cellular life with their large size (capsids can be up to 200 nm), intricate structures, and the presence of hundreds or even thousands of genes of which many appear to have been acquired from diverse cellular lineages [1–3]. Due to their large size, GVs are often not included in virome studies that apply a conventional size cutoff to define the “viral size fraction,” despite a growing recognition of their ubiquity, large diversity that can exceed that of bacteria and archaea [4, 5], importance as top-down regulators of eukaryotic host communities (e.g. demise of blooms) [6, 7] and drivers of evolution [8, 9]. Metagenomic analysis now allows the large-scale generation of GV metagenome-assembled genomes (GVMAGs) and has shown that their genomes are highly diverse and can encode genes for a wide range of functions including nutrient absorption, light assimilation, and nitrogen processing, as well as genes involved in glycolysis and the tricarboxylic acid cycle, together indicating their ability to modify essential aspects of their hosts metabolic processes [5, 10]. Recent global metagenomic surveys of marine viral biogeography revealed a heterogeneous distribution of GVs that was tightly linked to their hosts and highlighted the poles, particularly the Arctic Ocean, as a hotspot of unique viruses and GVs [11, 12]. The relevance of viruses in polar settings was proposed to be greater because of truncated marine polar food webs, as the pressure they exhibit is likely to be the main controlling biological factor on local microbial communities [13], a feature expected to be similar in other Arctic aquatic systems. Lakes are an essential part of the ecosystem of polar biomes and are integrators of their watersheds [14], therefore making them sentinels and indicators of local environmental changes [15]. Lakes integrate carbon and materials washed in from surrounding watersheds, contributing substantially to their integration into global biogeochemical cycles. Viruses in lakes play key roles as top-down controllers of primary producers and organic matter recycling [9, 16]. Polar lakes support rich microbial assemblages which form the basis for the truncated polar food webs [17], and GVs may play an especially significant role in these polar lakes where the low contribution of multicellular grazers allows microbes to dominate local food webs. Although polar lakes share many features, including oligotrophy, low temperatures, and extreme seasonality (i.e. continuous darkness during winter and continuous sunlight during summer), they can also vary substantially in salinity, stratification, and ice coverage. As such, polar lakes as a whole may represent an important reservoir of GV diversity. However, there is urgency to study polar lake virology as they are at risk of swift transformations due to global warming, especially in the High Arctic where lakes are disproportionately affected by the Arctic amplification [18]. The Last Ice Margin (LIM) area is situated on the northern coast of Ellesmere Island, Canada. This contiguous terrestrial ecosystem is protected and cooled by the adjacent marine Last Ice Area (LIA), characterized by the oldest and thickest ice in the Arctic Ocean, expected to persist longer in the LIA than in the rest of the Arctic Ocean [19]. Collecting data in such remote places remains extremely complex, expensive, and strongly limits the quantity and temporal resolution of sampling as well as in the volume of samples. Still, here we use our large dataset of metagenomes from the LIM and other Arctic lakes in a meta-analysis that aims to investigate for the very first time the diversity and similarities between GV populations from 20 lakes spread across Arctic, Antarctic, and temperate North American latitudes. First, we highlight that LIM lakes have highly endemic viral populations due to their remoteness and to the enduring cold conditions, and ensuing stability maintained by the LIA. Second, Arctic GVs are different from Antarctic ones due to distance and limited dispersal, despite sharing similar environmental drivers. Finally, polar (Arctic and Antarctic) GVs have a specific signature compared to non-polar (temperate) systems.

## Materials and methods

### Sampling from Arctic lakes

For this meta-analysis, four lakes from the LIM were studied and compared to Arctic, temperate, and Antarctic lakes. Data from the LIM lakes originate from the published studies of Lake A (82°35.48′N 75°15.57′W) [20, 21], Milne Lake (82°35.54′N, 80°35.76′W) [22], Thores Lake (82.65°N; 73.68°W) [23], and new data from Ward Hunt Lake (83°05.22′N; 74°08.72′W). Lake A is a highly stratified deep marine-derived meromictic lake with extreme variations of oxygen and salinity over depth. Milne Lake is an epishelf lake characterized by a freshwater layer that overlies marine water connected to the open ocean. It is believed to be one of the last epishelf lakes in the Arctic and suffered an important collapse of its ice shelf during the summer of 2020 [24]. Thores Lake is an ultra-oligotrophic large ice-contact proglacial lake dammed by Thores Glacier, a slow-moving, stable system on Ellesmere Island [23, 25].

Ward Hunt Lake is a polar freshwater lake that used to be perennially ice covered but has been experiencing sporadic ice-free summers since 2011 [26–28]. Three more Arctic lakes were sampled near the community of Resolute Bay (Qausuittuq, 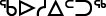) (74°41′N; 94°49′W) on Cornwallis Island, Canada (Meretta Lake, Char Lake and Resolute Lake). Finally, we used data from SAS2A Lake as a subarctic outgroup. SAS2A is a thermokarst lake located in Sasapimakwananistikw River Valley (SAS) near Whapmagoostui-Kuujjuarapik (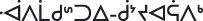) (55°13′N; 77°41′W), Canada. Although located at a relatively low latitude, SAS2A lies at the southern limit of the permafrost zone [29], a transition zone between temperate and polar latitudes.

All water samples were collected at multiple depths at all sites through bore holes through the ice, between July and August 2015 and 2019. Water was filtered onto filters of 0.02 μm pore size (or 0.2 μm and 0.02 μm) (Extended Data Table 1), from which DNA was extracted following Cruaud *et al*. [30] for 0.2 μm filters and Mueller *et al*. [31] for 0.02 μm. Sequencing was done on Illumina HiSeq / NextSeq.

### Meta-analysis data acquisition

To assess similarities or dissimilarities between our Arctic data and other lakes, we selected a set of 12 lakes that are characterized by different regimes of stratification, physico-chemical conditions, and ice cover at Antarctic and temperate latitudes. Genomic assemblies from temperate and Antarctic lakes were retrieved from publicy available studies in the Integrated Microbial Genomes and Microbiomes database (IMG/M). Antarctic lakes included four sites from the McMurdo Dry Valleys; Lake Bonney (77°43′S; 162°22′E), Lake Fryxell (77°37′S; 163°11′E), Deep Lake (77°34′S; 166°13′E), and Lake Vanda (77°31′S; 161°34′E) and four lakes from eastern Princess Elizabeth Land; Ace Lake (68°28′S; 78°11′E), Club Lake (68°33′S; 78°14′E), Organic Lake (68°27′S; 78°11′E), and Rauer Island Lake (68°33′S; 78°11′E). Temperate lakes included samples from Lake Croche (46°49′N; 72°30′W), Lake Montjoie (46°16.58′N; 57°07.59′W), Lake Simoncouche (48°13′N; 71°15′W) (Québec, Canada), and Lake Lanier (34°14′N; 83°57′W) (Georgia, USA).

### Assembly processing and GVMAG prediction

The resulting 374 metagenomes of both our own data and publicly available assemblies (see above) were processed per lake individually. BBmap (v.38.18) was used to map all reads against assembled contigs longer than 1000 bp [32]. Checking and potential correction of number of mismatch tags and sorting of bam files were done with samtools (v.1.6) synopsis “calmd -u” and “sort”. The bam alignments were used to generate a depth file with “jgi_summarize_bam_contig_depths”, for every sample and reconstruct genomes of minimum length of 75 000 bp from contigs (≥ 1500 bp) with metabat2 (v.2.15) binning. Generated bins were processed through the GVMAG identification and taxonomic classification tool GVClass (v. 0.9.3, https://github.com/NeLLi-team/gvclass) using default options. GVMAGs were identified and assigned to a phylum according to a conservative approach based on the consensus of single protein trees built from up to nine GVOGs (giant virus orthologous groups) (Extended Table 2): GVOGm0003, GVOGm0013, GVOGm0022, GVOGm0023, GVOGm0054, GVOGm0172, GVOGm0461, GVOGm0760, and GVOGm0890 [33]. To yield an assignment and taxonomic affiliation, all nearest neighbors in a GVOG phylogenetic tree must agree.

The viral nature of contigs composing all GVMAGs was then tested with the full geNomad pipeline with arguments “end-to-end --cleanup --splits 8”. One GVMAG assembled in Thores Lake was identified with an extreme length of > 20 Mb and was considered as a potential concatenation of similar individual GVMAGs. The bin was re-binned with a greater minimum score of edge (—maxS 98) to be split into more specific smaller GVMAGs with a minimum size of 75 000 bp. All resulting GVMAGs were kept for following statistical and geographical distribution analysis. Gene calling and protein clustering were performed with prodigal-gv.2.110 (https://github.com/apcamargo/prodigal-gv) and MMseqs2, respectively, with a protein clustering threshold of “--min-seq-id 0.5 -c 0.8” [34, 35].

### GVMAG species phylogenetic tree

All identified GVMAGs from all samples were pooled together and dereplicated with dRep dereplicate --ignoreGenomeQuality to avoid the CheckM scoring step as recommended by the user manual for virus genomes [36]. Dereplicated GVMAGs were added to the reference dataset of GV genomes from Aylward *et al*. [33]. A species tree was built with the New Simple Genome Tree (NSGTreev.0.4.0, https://github.com/NeLLi-team/nsgtree) computational pipeline. NSGtree was used with the set of phylogenetic markers GV0G7 (i.e. GVOGm0013, GVOGm0022, GVOGm0054, GVOGm0172, GVOGm0461, GVOGm0760, GVOGm0890). Markers were detected and filtered in query genomes using hmmsearch and hmmsearch_count_filter from hmmer (v.3.3.2). To build the species tree, only GVMAGs with at least four of the seven GVOGs and with no more than four copies of any of the GVOG7 were selected. A total of 1543 GVMAGs with assembly sizes ranging from 0.075 Mb to 2.126 Mb were retained. The produced tree was visualized with iTOL (v.6) [37]. Lineage down to the genus or subfamily level was assigned based on the GVClass taxonomic affiliation, in accordance with the recently proposed phylogenomic framework of the *Nucleocytoviricota* (i.e. *Algavirales*, *Asfuvirales*, *Imitervirales*, *Pandoravirales*, and *Pimascovirales*) [33]. Genome features like GC content (or guanine-cytosine content) and length were added to the tree.

### Geographic distribution of giant viruses

BBmap (v.38.18) was used to map reads to the 374 metagenomes with all dereplicated GVMAGs previously identified across the sampled lakes. Resulting alignment files were used to generate depth files using “jgi_summarize_bam_contig_depths” from metabat2 (v.2.15). Read depth coverage was calculated for each GVMAG based on the average read depth of each contig (GVMAG read depth coverage = sum (contig length × contig average total depth) / GVMAG total length). This GVMAG read depth coverage was then normalized by library size and multiplied by 10^9^ (i.e. average coverage per million reads). To account for reads mapping to shared genes across different GVMAGs, a GVMAG was only considered as “present” in a sample if at least 25% (for distance matrices and ordinations) or 70% of its bases (for presence/absence and dis/similarity analysis) were covered in this sample. The normalized and filtered (based on % bases covered) GVMAG coverages were then used to a build GVMAG sample-wide abundance table, for which the normalized filtered coverage was transformed with the Hellinger method (decostand{vegan} (v.2.5-7)) for distance-based analyses in R (v.4.1.1). Diversity and statistical analyses were performed in RStudio v. 1.4.1717. A Bray–Curtis dissimilarity matrix was calculated from the Hellinger-transformed tables to produce an ordination (metaMDS{vegan}) (v.2.5-7), which was visualized with a non-metric multidimensional scaling (NMDS) ordination. Effect of the sample region was tested with permutational multivariate analysis of variance (PERMANOVA) and was performed using adonis{vegan} (v.2.5-7) with 999 permutations. To show similarities/differences between sets of samples, tables were made binary to determine presence or absence of GVMAGs in samples (using the 70% cutoff of bases covered, see above), and UpSet plots were produced with the R package {UpSetR} (v.1.4.0). Upset plots display the number of total, unique and shared GVMAGs across lakes, and the four sampled regions (map_data{ggplot2}) (v.3.3.5). Unique fraction metric or unweighted UniFrac was used to compare the phylogeny of GVMAG communities between lakes and between regions. UniFrac distances were calculated from the 25% cutoff GVMAG sample-wide abundance table and the species phylogenetic tree (see above) using merge_phyloseq and UniFrac {phyloseq v.1.36.0}. The distance matrix was visualized with both NMDS (metaMDS{vegan}) (v.2.5-7) and a clustered dendrogram with hclust() {stats v.4.1.1} (agglomeration method = “mcquitty”) and dendro_data() {ggdendro v.0.1.23}.

### Eukaryotic diversity of LIM lakes

Since GVs infect eukaryotic hosts, we investigated the eukaryote populations in all four LIM lakes focusing on their ecological co-occurrence with local *Nucleocytoviricota*. We used BBMap v.38.93 and the SILVA SSU Ref NR 99 database 138 as a reference to extract 18S ribosomal RNA marker genes from reads [38, 39]. This was achieved through phyloFlash v.3.4 using default settings with the parameter “–almosteverything” and a 98% identity threshold for clustering [40]. The top hits’ last-common-ancestor consensus was used to determine an estimated taxonomic affiliation, and mapped read counts were used to provide an overview of community composition across samples. For consistency in reported taxonomic ranks, classifications were curated manually.

### Prediction of associative strength between GVMAGs and eukaryotes

We used Random Forest (RF) machine learning to determine if the presence of eukaryotic hosts could be predicted based on the giant virus community in a given sample. This RF approach allowed us to identify co-occurrence patterns using the transformed semi-quantitative data, which we interpreted similarly to classical correlation analyses. Each detected eukaryotic clade was treated as a target variable and transformed into a factor. To determine the number of variables selected at each split, we used tuneRF in the {randomForest v.4.7} function with ntreeTry = 1000, stepFactor = 1.5, improve = 0w.01. The quantification method for eukaryotes in the samples was changed from numerical counts to a custom qualitative categorization of “rare” or “abundant” based on whether the count in each sample was below or above the average count of all samples. This categorization was applied consistently to each sample. We analysed only the results from clades that had a relative abundance of at least 1% in at least one of the lakes and had an error rate below 25%. Associative strength between eukaryotic clades and *Nucleocytoviricota* order(s) was determined from mean decrease Gini index (i.e. a measure of the contribution of a variable to the homogeneity of the nodes and leaves in the resulting RF), with a higher score indicating greater importance of the variable in the model. For each model, we kept the viral family top explainer, plus up to three other families if their Gini index was within 80% of the one of the top explainer. An interaction network was built with Cytoscape v3.9.1 [41].

## Results and discussion

### Great and heterogeneous diversity of giant virus in lakes across latitudes

**Figure 1:**
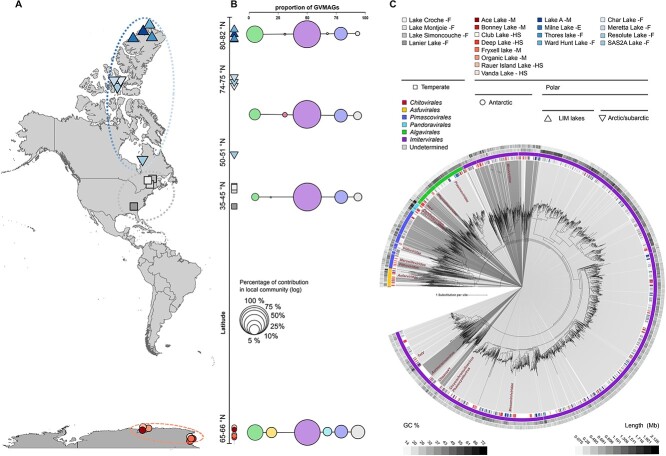
Metagenomic composition of lacustrine giant virus diversity from pole to pole; (A) geographic location of all 20 lakes used in our meta-analysis; lakes from the same region are identified with a similar color palette, and groupings are highlighted by dashed ellipses; Group indicators (“F,” “M,” “HS,” and “E”) following lake names stand for freshwater, meromictic, hypersaline, and epishelf, respectively; (B) distribution and contribution of giant virus orders across different group of lakes (from top to bottom: LIM lakes, Arctic/subarctic lakes, temperate lakes, and Antarctic lakes); bubble sizes are in log for visualization purposes; (C) maximum-likelihood phylogenetic tree of the GVMAGs inferred from a concatenated protein alignment of seven core GVOGs; shades indicate similar family level; cultivated viruses are indicated in red; tree annotations from inside to the outside: (1) GVMAG origin and cryo-feature (we considered the lake from which the GVMAG was initially assembled as its origin), (2) order classification, (3) GC content, and (4) GVMAG length.

We investigated the diversity of GVs from 374 metagenomes sampled across 20 different lakes in the Arctic (125 metagenomes), temperate (28 metagenomes), and Antarctic regions (221 metagenomes) (Fig. 1A, Supplementary Table 1). Using a custom pipeline (see Methods), we identified 3304 GVMAGs across all samples with a minimum horizontal coverage of 25% in at least one sample (i.e. > 25% of the genome length was mapped by reads).

Most GVMAGs (i.e. 2596) were assembled from temperate lakes (78.5%) and out of 709 GVMAGs assembled in polar lakes, 343 were assembled from Arctic lakes (10.4%) and 365 from Antarctic lakes (11.1%).

After comparison with already described GVMAGs in IMG, 90% of the 3304 identified GVMAGs were novel and out of the 10% redundant ones, 95% were identified in temperate lakes. We believe this significant difference in the number of GVMAGs assembled between biomes is multivariate. First, the diversity of eukaryotes is assumed to be higher in eutrophic temperate lakes [42] and could be reflected in a greater diversity of associated viruses. Second, polar lakes are cold, oligotrophic systems, and are expected to experience greater selective pressures and display more endemic diversity [43].

To examine the phylogenetic affiliation and community variation in GVMAGs between samples and regions, we built a *Nucleocytoviricota* species tree (Fig. 1C). The *Imitervirales* order dominated the communities in all regions and samples, with an average relative abundance of 76%. The *Algavirales* and *Pimascovirales* orders were, respectively, the second and third most abundant orders with average contribution of 8.9% and 6.0% across the entire dataset. *Asfuvirales*, *Chitovirales*, and *Pandoravirales* are depleted orders in all regions with low relative abundances of 1.9%, 0.9%, and 1.5%, respectively (Fig. 1B). Although the use of different filter sizes for multiple samples from the same lake and from one lake to another may introduce a slight bias in community diversity by favoring larger GV (*Imitervirales*) over smaller one (*Algavirales*), we believe that the concatenation of different filter sizes within lakes provides a concise description of punctual variations in community composition and shows that the distribution pattern observed is similar to community structures observed in marine environments [12].

Variations were observed in the GV community structure across regions. In particular, the maximum relative contribution of *Imitervirales* was found in temperate lakes (85.1%), while they were slightly less abundant at high latitudes (61.6% and 74.4% in Antarctic and Arctic, respectively) (Fig. 1B). *Algavirales* showed variations in relative abundance along latitude. Their highest abundance was observed in LIM lakes (14.3%) and Antarctic lakes (12.8%). Their relative abundance was lower in Arctic/subarctic (5.5%) and temperate lakes (3.7%). A similar pattern of higher *Algavirales* contribution at polar latitudes compared to temperate regions was also observed for marine viruses [12]. *Asfuvirales* and *Pandoravirales* were more abundant in Antarctic lakes, while *Chitovirales* were more abundant in Arctic/subarctic lakes. *Pimascovirales* was the only order showing no evidence of latitudinal variation (i.e. average relative abundance 6% ± 1.1). Interestingly, Antarctic lakes presented the largest relative abundance of GVMAGs for which taxonomy could not be confidently assigned at lower ranks by GVClass (8.88%) compared with only 3.1%, 3.5%, and 1.9% in Arctic/subarctic, Temperate, and LIM lakes, respectively. However, some of these “undetermined” GVMAGs branched within the *Pandoravirales*, *Algalvirales*, or *Primascovirales* orders in the species phylogenetic tree, suggesting they may represent new clades within these orders.

**Figure 2:**
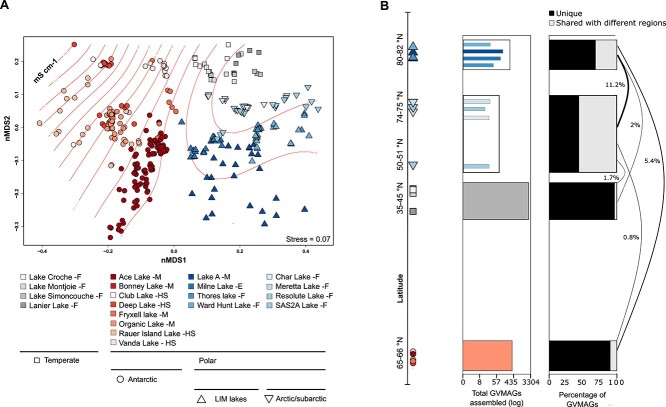
Distribution and similarities in giant virus communities across lakes and regions; (A) NMDS ordination of lake metagenomes; lakes from the same region are identified with common shapes and a similar color palette; group indicators (“F,” “M,” “HS,” and “E”) following lake names stand for freshwater, meromictic, hypersaline, and epishelf, respectively; sample conductivity is represented with red lines on the ordination diagram; (B) horizontal bar plot showing the distribution of all assembled GVMAGs across all regions (left) and the percentage of unique and shared GVMAGs across regions (right); only percentages of shared GVMAGs >0.1% between two regions are shown.

We next compared all GVMAG communities of all samples from all regions in an NMDS ordination using the Bray–Curtis dissimilarity metric. Our results suggest that GVMAG assemblies from the same study regions are ordinated closer together (Fig. 2A). Some of the lakes in the dataset display a wide range of salinity across their water column, and similar conductivity can be measured in diverse lakes from different regions (i.e. similar salinity levels in Lake A and Ace Lake or between Cornwallis lakes and temperate lakes). To address the potential impact of salinity on structuring GV communities and driving dissimilarities between samples, we modeled sample conductivity on the ordination plot (Fig. 2A). Our results show that GV communities of similar conductivity samples from different lakes did not group together and clustered with samples from the same lake and same latitude first (Fig. 2A). This substantiates that lakes can be considered as units in this study and suggests that salinity may not be the major environmental variable that drives GVMAG community structure and that the variation among communities could mainly be a result of speciation occurring along latitudinal gradients.

### *Nucleocytoviricota* in the LIM and overlap with Arctic/subarctic lakes

**Figure 3:**
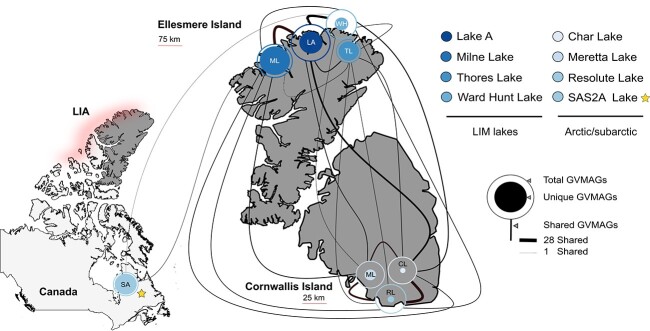
Structural differentiation of giant virus communities across and between LIM lakes and Arctic/subarctic lakes; location of LIM lakes and Arctic/subarctic lakes; the map shows the numbers of total, unique, and shared GVMAGs across the lakes; letters within the circles correspond to the initials of the lake names; the star indicates SAS2A as the subarctic outgroup (see Methods); the LIA is represented by a shading on the north coast of the archipelago and Ellesmere Island; the LIA is shown in a schematic style here to emphasize its proximity to the LIM lakes.

We showed that GV communities of Arctic lakes are unlike that of lakes from other regions identified in this study (Fig. 2A and B). Because of the unique features of lakes in the LIM, which is expected to remain cool and become the last northern inland refuge for ice-dependent species in the coming century thanks to the enduring LIA, we further explored the *Nucleocytoviricota* inter-overlap between LIM lakes and intra-overlap with Arctic/subarctic lakes using a presence/absence approach (see Methods). All four sampled lakes in the LIM showed more than 50% of uniqueness in their GV communities (Fig. 3). Milne Lake and Thores Lake were the lakes showing the two most unique communities with 70.75% and 72.97% of uniqueness rate, respectively (Fig. 3, Extended Fig. 1). Ward Hunt Lake and Lake A have slightly smaller unique communities with 51.85% and 66.87% of exclusive GVMAGs and were also the lakes that shared the highest number of GVMAGs with each other, 42.59% and 14.64% respectively. The greater overlap could be a result of their direct proximity and their more similar watershed compared to the other lakes (Fig. 3, Extended Fig. 1). Still, Thores Lake seemed to be the most dissimilar lake of all (Extended Fig. 1). Geographic distance from the ocean and the origin of cryosphere (terrestrial vs. marine) might be the potential drivers of dissimilarities in the *Nucleocytoviricota*, with Thores Lake being fed by glacier meltwater and runoff with a distinct climate relative to the other lakes under greater marine influence [23]. Lakes from the LIM and Arctic/subarctic regions were the most connected in our dataset (i.e. sharing 11.2% of their GVMAGs) (Fig. 2B) notably with lakes Meretta, Char, and Resolute. Located on the southern coast of Cornwallis Island, all three lakes shared 11.76% of all GVMAGs identified across them all and showed only 23.52%, 12.94%, and 18.23% of unique GVMGAs, respectively (Fig. 3). The greater number of GVMAGs common to all three lakes was expected as they have very similar watersheds and both Meretta and Char outflow into Resolute Lake. We noted that all lakes from the LIM have at least one shared GVMAG with at least one of the three Cornwallis lakes. Our current dataset does not allow us to characterize the direction or the nature of interactions of these shared GVMAGs, and these could be the consequence of convergent evolution or the infection of a widespread host species. However, recent changes in the limnological properties and ecology of lakes in the LIM could also explain such similarities between both regions. The impact of climate change in the LIM has been dramatic. During the summer of 2020, the Milne Ice Shelf broke off in a calving event that resulted in the loss of the epishelf lake and its viral assemblages with distinct genetic repertoires with it [20, 24]. Many lakes in the LIM are showing non-linear changes in response to climate change; increase in the size of moats on the shores, decrease in ice-cover thickness and durability and variation in conductivity [44]. The first complete disappearance of summer ice on Ward Hunt Lake was reported in 2011, resulting in a very different ecosystem [28]. Such alterations can strongly alter the lakes, making them less autotrophic, warmer, and with weaker stratification, some changes that could increase rapid growth of protists correlated with an increased viral diversity, viral lytic activity, and viral shunt.

Recently, sequences of non-local plant species were identified in Thores Lake and were hypothesized to be the result of wind-blown pollen transport [23]. An intensification of airborne seeding in response to the absence of protective ice cover and the northward migration of species could facilitate increased exchanges between GVs from LIM lakes and other Arctic/subarctic lakes [45]. Also, mimiviruses and other giant viral particles are often enveloped in virus-encoded sugars that adhere to various organisms through glycoside interactions, including flying arthropods, leading to potential zoochory events [46]. The construction of GV dispersal mechanisms, the isolation, and detailed study of shared GVs could enable the tracking of viral migration patterns and the identification of sentinel species for shifts in the environmental conditions of lakes. The outgroup, SAS2A Lake, contained a high number of unique GVs (88.5%) and shared only three GVMAGs with the communities in LIM lakes (Fig. 3). Nonetheless, SAS2A communities clustered together with the other Arctic and subarctic GV communities in the NMDS ordinations, despite their considerable dissimilarity and greater complexity compared to these (Fig. 2A). This suggests the presence of geographical factors that influence the viral composition of these lakes in ways that have not yet been identified.

### Giant virus diversity and eukaryotic interactions in LIM lakes

**Figure 4:**
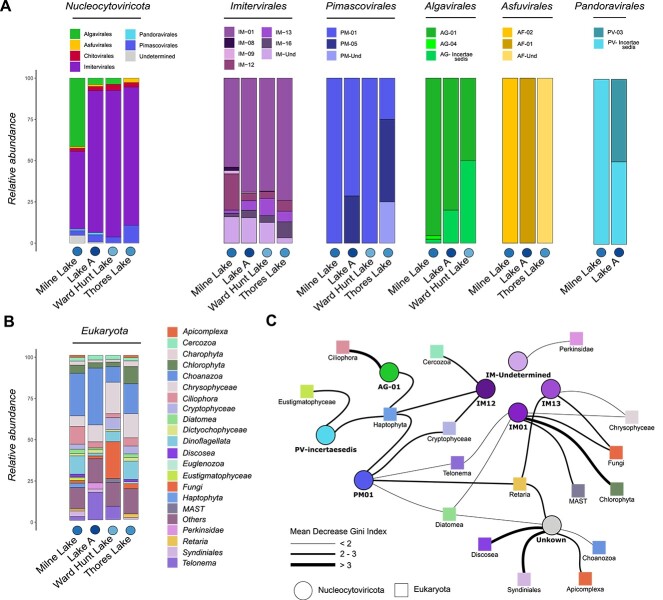
Giant virus community composition across LIM lakes and putative eukaryotic hosts; (A) relative abundance of giant virus orders and families in each LIM lake; (B) relative abundance of eukaryotic clades in each LIM lake; (C) network of predicted strength of association between giant virus families and eukaryotic clades in LIM lakes; the thickness of interaction links between groups is related to the decreasing Gini index; a thicker link stands for a higher score, which indicates greater importance of the variable in the model, and stronger predicted associative strength (see Methods for more details), circles represent giant virus families, and squares represent eukaryotic groups; the color legend is the same as in A and B.

Comparing *Nucleocytoviricota* taxonomy across LIM lakes, Thores Lake was the only one that had a complete absence of *Algavirales* and was along with Lake A and Ward Hunt Lake dominated by *Imitervirales* (> 80% in all three) (Fig. 4A). Milne Lake was co-dominated by *Imitervirales* and *Algavirales*, representing 47.16% and 41.50% of its community, respectively. The significant difference in *Algavirales* proportions between Milne and Thores suggests that the proximity to the ocean along with the watershed type (marine versus glacial input sources) might alter the *Nucleocytoviricota* composition of these lakes.

Strong dissimilarities between lakes were also observed at family-level taxonomy assignments of GVs. Milne showed greater richness within the IM-12 family (*Allomimiviridae*) than any other lake (Fig. 4A). Some members of the IM-12 family are known to infect *Tetraselmis* sp., a green algae found in both marine and freshwater ecosystems [47]. This observation is consistent with the high proportion and diversity of *Algavirales* (Fig. 4A) in the lake and likely reflects the algal host diversity and availability in the Milne water column and its connection to seawater. Other lakes showed notable changes in their *Nucleocytoviricota* community*.* Thores Lake was distinguished by its higher proportion and diversity of *Pimascovirales* (10.8%) and IM-16 (*Mimiviridae*) (9.4%) (Fig. 4A). Finally, Ward Hunt Lake showed the highest abundance of *Imitervirales* IM-13 family and total absence of *Asfuvirales* representatives.

Given that viruses are dependent on host organisms for replication, it is expected that the diversity and population of viruses will be closely linked to the availability and variety of potential host populations (as reviewed in Schulz *et al*. [48]). To evaluate potential co-occurrence between GVMAGs and candidate hosts, we used RF to derive “potential interaction indexes” (see Methods) between eukaryotic clades from lakes (Fig. 4B) and GV families. This analysis underscored some expected and known interactions, such as a strong co-occurrence between *Haptophyta* algae and PV-incertae-sedis, IM-12, AG-01, and PM-01 (Fig. 4C). Haptophytes, especially *Prymnesiophyceae*, are known for their frequent blooms. *Emiliania huxleyi*, the most abundant calcifying haptophyte in oceans [49], is a key player in global biogeochemical cycles [50, 51] and is susceptible to *E. huxleyi* virus, a *Pandoravirales* (PV) [33], which has a significant impact on these cycles. In LIM lakes, the coexistence of these groups hints at similar interactions and potential local biogeochemistry variations. Haptophytes have also been reported to be infected by *Imitervirales* like *Phaeocystis globosa* virus and *Chrysochromulina parva* virus [52–54], and *Algavirales* endogenization has also been observed. No haptophyte is currently known to be susceptible to PM-01 virus or *Pimascovirales* and the interaction link we showed could also be a co-occurrence between these groups. Other known host–virus interactions were identified in our results. We showed a strong correlation between *Chlorophyta* and the IM-01 family. The IM-01 (*Mesomimiviridae*) have members with native hosts from *Chlorophyta* (i.e. *Pyramimonas orientalis* virus and its *Prasinophyceae* host) [48, 55], and integrate genomes into the host chromosome [10, 48, 56]. Interestingly, *Chlorophyta* are also known to be native hosts of giant viruses from *Algavirales* and AG-01 family [56–58]; however, we were not able to discern this connection, suggesting that our analysis is probably limited by an uneven distribution of certain taxonomic groups or other factors. Beyond these known interactions, we identified several unexpected virus-eukaryotes pairs with high specificity to certain lakes and/or samples. Ward Hunt Lake eukaryotic population was dominated by Fungi in our dataset and showed specific high abundance of IM-13 (Fig. 4B), which were accordingly linked in our interaction network (Fig. 4C). Fungi have previously been predicted to be potential hosts for *Imitervirales* and notably IM-01 viruses [5, 59], a link also found in our network. However, this must be considered with caution as many aquatic Fungi are known to be parasitic to numerous algal species (e.g. *Chrysophyceae*, *Chlorophyceae*) and correlation between these groups could also be a co-occurrence in response to the same host. Another unexpected link was observed between alveolate *Ciliophora* and AG-01 (Fig. 4C). This interaction is to be taken with caution as it appears unusual considering that *Algavirales* are known to infect “algae”, while *Ciliophora* are heterotroph *Alveolata*. However, signs of endogenization of viruses from the *Algavirales* order have been identified in alveolates [5] and ciliates may be associated with green algal symbionts. A studied example of such a tripartite association is *Paramecium bursaria Chlorella* virus, which infects a symbiotic *Chlorella* within *P. bursaria* [60, 61]. We could therefore envision a similar interaction through the connection shown in our results.

Overall and despite some limitations, our approach provides the foundation for targeted sampling efforts that will allow experimental verification of predicted interactions.

### Oceans as potential driver of sporadic interactions between poles

Out of the total shared GVMAGs (34) between poles (Fig. 2B), 79.41% were assembled from Milne Lake. The majority of them (85.2%) was shared between Milne Lake and Ace Lake only. Ace Lake is a saline (i.e. avg: 52.9 mS cm^−1^) meromictic lake, much more similar limnologically to the LIM Lake A, a meromictic lake with similar saline deep waters (i.e. avg: 45.8 mS cm^−1^) (Supplementary Table 1). Both display an upper aerobic mixolimnion and an anaerobic monimolimnion [20, 62], while the entire water column of Milne Lake is oxic and less saline [20]. However, only three shared GVMAGs were identified between Ace Lake and Lake A in our study.

We investigated the taxonomic affiliation of Milne-Ace shared GVMAGs and identified that most of them (91.66%) belong to the *Algavirales* order and to the AG-01 family (*Prasinoviridae*), a family known to infect marine phytoplankton and notably *Micromonas pusilla,* which, unlike most marine algae, is distributed widely in both temperate and polar waters [63–65]. These results were consistent with our past study where we showed an abundance of *M. pusilla* virus SP1-related viruses at the freshwater/seawater interface in Milne Lake [22], corresponding to a higher abundance of *Micromonas* in the same samples. The connection of Milne Lake to the Arctic Ocean represents a potential way in which lakes can interact and exchange with marine systems, leading to global dispersal of giant viruses. Ace Lake, as it exists today, is meromictic; however, its palaeolimnology shows a gradual transformation of the original freshwater into a marine-like basin with typical marine plankton, which eventually became completely isolated by isostatic rebound ~5100 years BP [62]. Located < 200 m away from Long Fjord, the nearest marine inlet, the two bodies of water are separated only by a sill at ~2 m above the lake [66, 67]. Considering this proximity, it is possible that some infrequent exchanges occurred between the two water bodies notably over marine spreads and wind transportation. The greater proximity of Ace Lake to the ocean and its lack of ice cover could explain why more GVMAGs are shared with Milne Lake than with Lake A, which is ice-covered and not directly connected to the ocean. Further investigations should be led in this direction, and the recently generated polar/non-polar marine GVMAGs would be a relevant comparison point to search for overlap with marine data and potential role of oceans in interactions between polar lakes [68].

Nonetheless, this interaction between Ace Lake and Milne Lake is atypical, while our results highlight a strong endemic signal between Arctic and Antarctic lacustrine GVs (Fig. 2B). We posit that geographic isolation among the lakes in our dataset has driven GVs to adapt to their distinctive environments, resulting in an important reservoir of diverse and specialized endemic viruses. These viruses may play crucial roles in local biogeochemical processes, offering opportunities for further investigations and discoveries.

### Barrier and endemism of GVMAGs between polar and temperate regions

**Figure 5:**
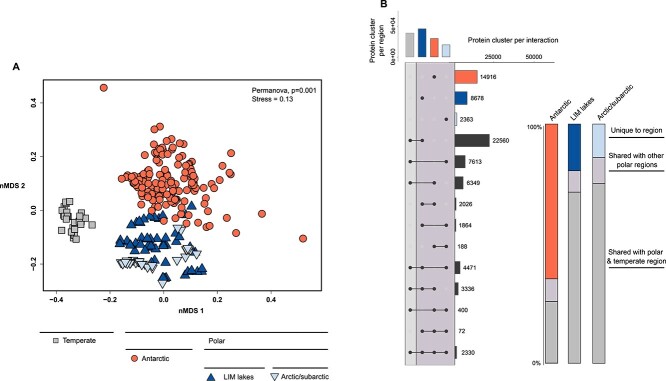
Giant virus phylogeny and genetic makeup dissimilarities between polar and temperate lakes; (A) NMDS ordination of phylogenetic distances (Unifrac) between GVMAGs from all samples; lakes from the same region are identified with a common shape and color; (B) UpSet plot showing the numbers of unique polar protein clusters and shared between polar and temperate regions; bar plot on the right shows proportions of proteins clusters unique to a polar region, shared with other polar regions and shared with temperate and polar regions.

Using the previously constructed phylogenetic tree (Fig. 1C), we computed an NMDS based on the phylogenetic distances across samples (unweighted Unifrac) to measure genomic relatedness of high confidence GVs across regions. Compared with an ordination based on abundance of GVMAGs (Fig. 2A), this NMDS based on phylogenetic distance enabled us to investigate (dis)similarity between lakes, GV communities on the basis of evolutionary relationships. Significant differences in community composition were detected among all categories (PERMANOVA, *P* = 0.001), and the analysis showed that GVMAGs are primarily clustered according to biome type before being grouped by poles within the polar cluster (Fig. 5A). These results are consistent with our previous observations (Fig. 2A) and confirm the existence of a strong phylogenetic dissimilarity between the GV communities of temperate and polar lakes. We investigated the overlap and uniqueness of GVMAGs between regions using the previously described presence/absence method. Temperate lakes showed the highest percentage of uniqueness in their GV communities, with 96.5% of endemic viruses (Fig. 2B). Interestingly, among the 3.5% of GVMAGs shared with other regions, 0.2% were shared with Antarctic lakes and 3.2% with Arctic lakes. Although minimal compared to the endemism rate of temperate viruses, it is interesting to show this higher percentage of overlap with Arctic lakes, an observation most likely due to the greater geographic proximity of these lakes. However, LIM lakes shared many more GVMAGs with Antarctic lakes than with the temperate ones regardless of geographic distance (Fig. 2A and B). This implies the existence of other intrinsic factors that contribute to the unique characteristics of polar communities and suggests the existence of an ecological barrier separating temperate and polar lakes similar to the one recently shown in marine ecosystems [68]. In their study, Meng *et al*. [68] identified numerous viral genes associated with polar adaptation and notably to cold environments by changing their functional repertoire. They noted that most of the homologues of these viral genes were not identified as polar-adaptive genes in their eukaryotic hosts, suggesting that viral evolutionary strategy was independent of the polar adaptation of their potential hosts.

In polar lakes, where spatial isolation (i.e. distance and lake ice) leads to rare to no interactions, local GVs have likely undergone similar speciation and evolutionary processes. To investigate differences in gene content between polar and temperate GVMAGs, we performed an all-vs-all protein clustering and analyzed the global sample distribution of the resulting protein clusters (Fig. 5B). Most protein clusters were only found in temperate GVMAGs, due to the higher number and larger diversity of GV assembled from temperate lakes. However, despite a very large database of temperate GVMAGs, we still showed that a significant proportion (~20% Arctic, ~60% Antarctic) of protein clusters were specific to polar regions (Fig. 5B). The higher proportion of protein clusters in the Antarctic compared to the one in the Arctic suggests that we have not yet sampled enough of the northern lakes to find all “accessory” genes that might be specific to Arctic environments. Nevertheless, our results highlight the genetic content of GVs as a potential driver of dissimilarities between regions and suggest that the genetic make-up of GVs may reflect adaptation to local environmental pressures, possibly associated with the patterns of strong endemism. The results were near-identical when restricting the analysis to contigs predicted as viral by geNomad instead of the entire GVMAGs, suggesting that the trends observed were truly driven by genes encoded on giant virus genomes (Extended Fig. 2).

## Conclusions and perspectives

Giant viruses likely have a crucial impact on biogeochemical cycles, can modulate host evolution and, thus, play a role in their ecosystem functioning. However, very little is known about their diversity and richness in lakes, especially in polar lakes where we believe them to have higher contribution as top-down controllers in the truncated polar food webs. Here we used our large database of lake metagenomes from the Canadian Arctic and subarctic latitudes to lead a first survey and meta-analysis of lacustrine GV diversity and distribution from 20 lakes from pole to pole. To do so, we combined recent tools for GV genome identification, phylogenomics, GV gene calling, and all-against-all genome mapping to perform a state-of-the art meta-analysis. We assembled 3304 GVMAGs across all lakes and showed these ecosystems as untapped reservoirs of GV diversity. Phylogenomic analysis inferred from a concatenated protein alignment of seven core GVOGs indicated that GVs from our meta-analysis were dispersed across all *Nucleocytoviricota* orders with a predominance of *Imitervirales* representatives. A high degree of endemism of GV populations and their genetic content was demonstrated between lakes in the same region and between lakes in the same biome (Antarctic and Arctic polar lakes). Finally, we identified the existence of a strong polar/temperate barrier between lacustrine GV populations, like the one recently identified in marine ecosystems. Ultimately, we have established a unique genomic reference database of GVs from polar lakes. This resource opens up fresh avenues for targeted research into viral polar genetic adaptations, co-evolution, and enhances our understanding of GV ecology in polar lakes—important sentinels of Earth’s climate.

## Supplementary Material

Extended_FIG_1_ycae048

Extended_Fig_2_ycae048

Extended_Data_table_1_ycae048

Expended_Data_table_2_ycae048

Supplementary_Table_2_ycae048

## Data Availability

The data underlying this article were accessed from IMG/VR database. Unique identifiers SRA for metagenomes are listed in Extended Data Table 1. The phylogenetic tree is available with figshare DOI: 10.6084/m9.figshare.25000571. GVMAGs sequences are available under Bioproject PRJNA1074374 and figshare DOI: 10.6084/m9.figshare.25000571. Complementary data to Lac A metagenomes such as raw amplicon sequences and bacterial bin files are available under Adrien Vigneron *et al*., Bioproject PRJNA616293.
