# Supplementary material for: Distinct and rich assemblages of giant viruses in Arctic and Antarctic lakes
Source: ISME Commun. 2024 Mar 29;4(1):ycae048. doi: 10.1093/ismeco/ycae048 (PMC11128243; doi:10.1093/ismeco/ycae048)
Supplement: Extended_FIG_1_ycae048 [file extended_fig_1_ycae048.pdf]

Extended Data Fig. 1: Giant virus community similarities across LIM lakes

Dendrogram showing hierarchical cluster analysis of giant virus communities found in lakes from the Last Ice Margin.

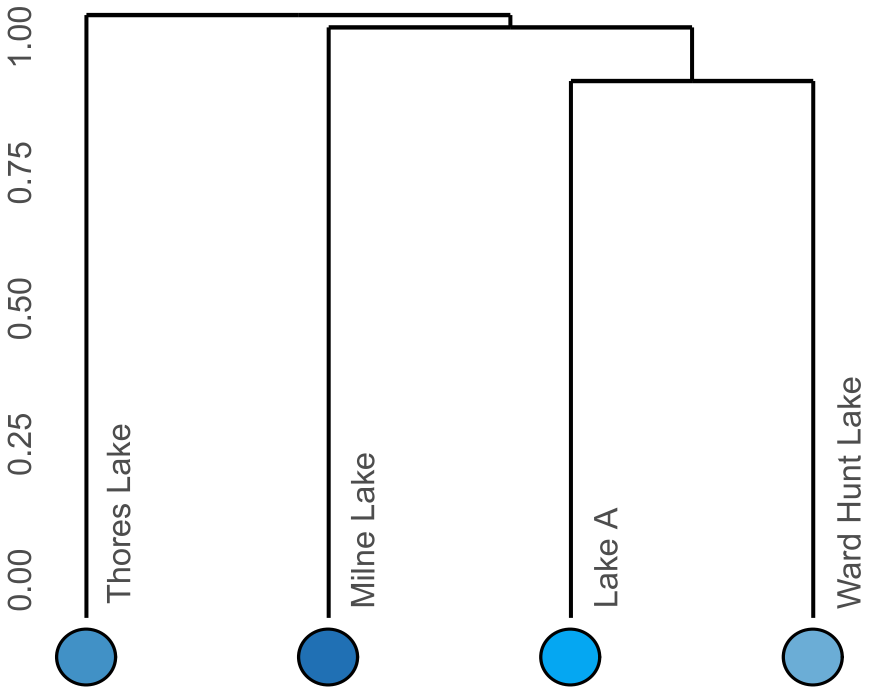
1.00
0.75
0.50
0.25
0.00
Thores Lake
Milne Lake
Lake A
Ward Hunt Lake
