# Supplementary material for: Distinct and rich assemblages of giant viruses in Arctic and Antarctic lakes
Source: ISME Commun. 2024 Mar 29;4(1):ycae048. doi: 10.1093/ismeco/ycae048 (PMC11128243; doi:10.1093/ismeco/ycae048)
Supplement: Extended_Fig_2_ycae048 [file extended_fig_2_ycae048.pdf]

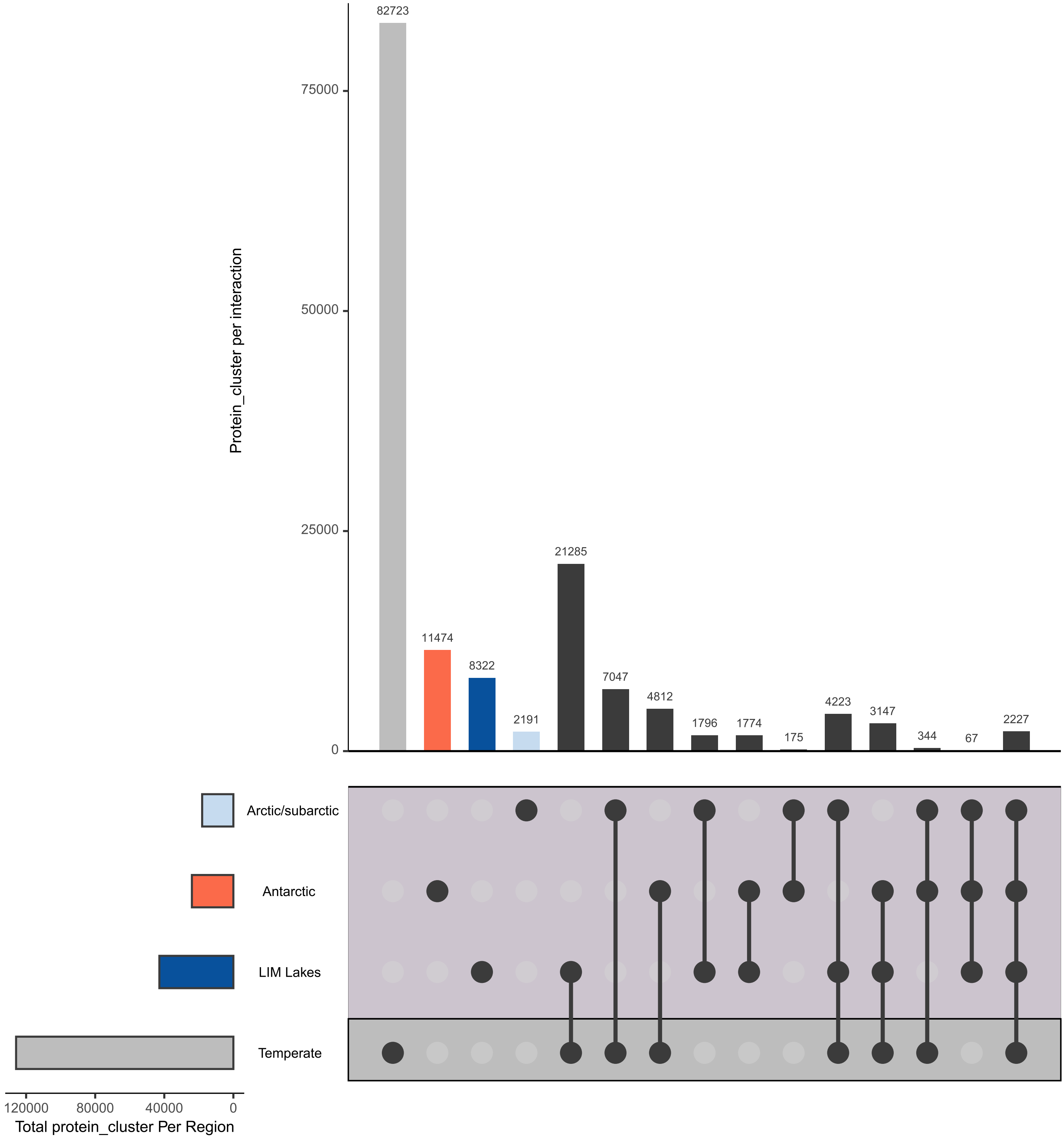

82723
11474
8322
2191
21285
7047
4812
1796
1774
175
4223
3147
344
67
2227
75000
50000
25000
0
Protein_cluster per interaction
Arctic/subarctic
Antarctic
LIM Lakes
Temperate
120000
80000
40000
0
Total protein_cluster Per Region

Extended Data Fig. 2: Giant virus genetic makeup dissimilarities between polar and temperate lakes

Functional potential diversity analysis restricted to contigs predicted as viral by geNomad instead of the entire GVMAGs. The UpSet plot shows the numbers of unique polar protein clusters and shared between polar and temperate regions.
