# Supplementary material for: Distinct and rich assemblages of giant viruses in Arctic and Antarctic lakes
Source: ISME Commun. 2024 Mar 29;4(1):ycae048. doi: 10.1093/ismeco/ycae048 (PMC11128243; doi:10.1093/ismeco/ycae048)
Supplement: Expended_Data_table_2_ycae048 [file expended_data_table_2_ycae048.pdf]

GVOG ID	Name	Annotation
GVOGm0003	MCP	Major capsid protein
GVOGm0013	SFII	Superfamily II helicase
GVOGm0022	RNAPS	Small RNA polymerase subunit
GVOGm0023	RNAPL	Large RNA polymerase subunit
GVOGm0054	PolB	Family B DNA Polymerase
GVOGm0172	TFIIB	TFIIB transcriptional factor
GVOGm0461	TopoII	Topoisomerase family II
GVOGm0760	A32	A32-like packaging ATPase
GVOGm0890	VLTF3	Virus late transcription factor 3

Extended Data table. 2: Identity and annotation of Giant virus Orthologous groups GVOGs used for taxonomy assignation.
